# Defining “Adverse Environmental Impact” and Making § 316(b) Decisions: A Fisheries Management Approach

**DOI:** 10.1100/tsw.2002.191

**Published:** 2002-05-17

**Authors:** David E. Bailey, Kristy A.N. Bulleit

**Affiliations:** Mirant Corporation, 8711 Westphalia Road, Upper Marlboro, MD 20772, USA; Hunton & Williams, 1900 K Street, N.W., Washington, D.C. 20006-1109, USA

**Keywords:** entrainment, impingement, 316(b), adverse environmental impact, fishery, survival, intake technology, costs and benefits, maximum net benefit, cooling water, intake structure

## Abstract

The electric utility industry has developed an approach for decisionmaking that includes a definition of Adverse Environmental Impact (AEI) and an implementation process. The definition of AEI is based on lessons from fishery management science and analysis of the statutory term “adverse environmental impact” and is consistent with current natural resource management policy. The industry has proposed a definition focusing on “unacceptable risk to the population’s ability to sustain itself, to support reasonably anticipated commercial or recreational harvests, or to perform its normal ecological function.” This definition focuses not on counting individual fish or eggs cropped by the various uses of a water body, but on preserving populations of aquatic organisms and their functions in the aquatic community. The definition recognizes that assessment of AEI should be site-specific and requires both a biological decision and a balancing of diverse societal values. The industry believes that the definition of AEI should be implemented in a process that will maximize the overall societal benefit of the § 316(b) decision by considering the facility’s physical location, design, and operation, as well as the local biology. The approach considers effects on affected fish and shellfish populations and the benefits of any necessary best technology available (BTA) alternatives. This is accomplished through consideration of population impacts, which conversely allows consideration of the benefits of any necessary BTA modifications. This in turn allows selection of BTAs that will protect potentially affected populations in a cost-effective manner. The process also employs risk assessment with stakeholder participation, in accordance with EPA’s Guidelines for Ecological Risk Assessment. The information and tools are now available to make informed decisions about site-specific impacts that will ensure protection of aquatic ecosystems and best serve the public interest.

### INTRODUCTION

Generating electric power requires cooling water to condense steam after it is used in steam-powered turbines. Withdrawing cooling water from surface waters for this purpose can impinge fish on screens and entrain fish and shellfish, eggs, and larvae. Impingement is the entrapment of fish or shellfish on screens that are used to prevent condenser blockage. Entrainment is the passing of organisms through the cooling water system, which may cause mortality from exposure to heat, physical stress, or chemicals.

In § 316 of the Clean Water Act, Congress included a subsection (a) to allow variances from thermal standards, if it is demonstrated that there will be "protection and propagation of a balanced, indigenous population of shellfish, fish and wildlife in and on the waterbody." Immediately following is § 316(b), which states that any standard applicable to a point source under § 301 or § 306 of the Act "shall require that the location, design, construction, and capacity of cooling water intake structures reflect the best technology available for minimizing adverse environmental impact." The U.S. Environmental Protection Agency (EPA), driven by a lawsuit in federal district court in New York State, is conducting a rulemaking to implement § 316(b)[1].

The purpose of this paper is to contribute to the development of § 316(b) regulations that will both protect living aquatic resources and reflect sound social policy. It addresses the following topics:

- The history of § 316(b) and EPA's current approach to the rulemaking
- The need for a definition of "adverse environmental impact"
- The need for a rule based on the tools and principles of fisheries management science
- The need for a rule that maximizes net social benefit
- A suggested approach that meets these needs.

### A BRIEF HISTORY OF § 316(B) AND EPA'S 316(B) RULEMAKING

Congress enacted § 316(b) of the Clean Water Act in 1972. The language of § 316(b) first appeared in the Conference Report on the 1972 Federal Water Pollution Control Act Amendments in a section called "Thermal Discharges." There was no comparable language in earlier House or Senate bills and little testimony or debate in the record explaining its sudden appearance. It appears, in fact, to have been an afterthought[2].

In December 1973, little more than a year after the statute was enacted, EPA proposed a rule to implement § 316(b). The rule was finalized in 1976. Both the proposed and final versions referenced EPA Development Documents, which described factors and design alternatives to consider when making a § 316(b) determination. A preamble to the 1976 final rule said that "decisions relating to the best technology available are to be made on a case-by-case basis." The rule was short-lived, for the Fourth Circuit Court of Appeals set it aside on procedural grounds.[1] In 1977, EPA published a draft guidance document, but this was never finalized[2,3]. For over 20 years, § 316(b) has been widely implemented on a site-specific basis, guided by the 1977 draft guidance rather than by regulations.

In 1993, several environmental groups filed suit against EPA in a U.S. district court in New York, seeking to compel EPA to issue regulations to implement § 316(b).[2] EPA and the environmental plaintiffs settled the case and agreed to a rulemaking schedule in a consent agreement entered by the court.

EPA's final rule for *new* facilities was published in the Federal Register, December 18, 2001, and a new proposed rule for *existing* facilities was published April 9, 2002. Although new and existing facilities do deserve different treatment under § 316(b), many issues raised by the proposed new facilities rule will be the same as or similar to the issues for existing sources.

### THE NEED FOR A DEFINITION OF "ADVERSE ENVIRONMENTAL IMPACT"

In the "Phase I" rulemaking for new facilities, EPA reports that it has received numerous comments addressing how "adverse environmental impact" (AEI) should be defined[4]. A definition is important because it establishes the basis for resource protection and provides a standard for selecting best technology available (BTA), in cases where BTA is required.

While a number of possible definitions of AEI have been offered, the following definition, proposed by the Utility Water Act Group (UWAG), is both scientifically sound and socially relevant for § 316(b) decisionmaking: "Adverse environmental impact is a reduction in one or more representative indicator species that (1) creates an unacceptable risk to the population's ability to sustain itself, to support reasonably anticipated commercial or recreational harvests, or to perform its normal ecological function and (2) is attributable to the operation of the cooling water intake structure"[5].

This definition focuses on protection at the population level. As stated in AFS Policy Statement #1, a goal of fisheries management is "to ensure self-sustaining populations that would support commercial and recreational fishing

[1] *Appalachian Power Co. v. Train*, 566 F.2d 451, 459 (4th Cir. 1977).

[2] *Cronin v. Browner*, 898 F. Supp. 1052 (S.D.N.Y. 1995).

both now and in the future"[6]. As Suter and Barnthouse concluded, "(t)he reproducing population is the smallest ecological unit that is persistent on the human time scale, and hence the lowest level that we can meaningfully protect"[7].

Despite this emphasis on population-level effects, it is recognized that for species whose populations are at critically low levels, the population can become endangered, in which case the protection of individual organisms through the Endangered Species Act[3] is appropriate. In addition to the federal statute, many states have enacted similar endangered species legislation.[4] These statutes, already in place, should and will be applied no matter what § 316(b) regulatory process EPA ultimately adopts.

The proposed AEI definition set out above also acknowledges that ecosystem integrity, structure, and function must be protected and, from a fisheries management perspective, that reasonably expected harvests should not be impaired. Finally, the recommended definition of AEI incorporates the idea of risk and therefore invokes risk management as part of the AEI decisionmaking process.

### THE NEED FOR A RULE BASED ON THE TOOLS AND PRINCIPLES OF FISHERIES MANAGEMENT SCIENCE

The effect of cooling water intake structures (CWIS) on fisheries is fundamentally similar to the effects of recreational and commercial harvesting of fish and associated effects of bycatch and bait collection. One primary difference is which species are affected. Fishery harvesting, of course, targets species that are desirable for human or animal food consumption and sport interest, while CWIS losses are a function of the interaction of fishery populations with the CWIS. CWIS vulnerability tends to be highly variable, depending on the CWIS location, design, and species' life history and behavior. Nevertheless, the similarities between losses from fishing and CWIS losses are such that CWIS effects on the fishery can be evaluated using the same basic approaches used by state and federal fishery managers to manage their commercial and recreational fisheries. The species and sizes of fish and shellfish impinged and entrained can be quantified and evaluated in the context of fishery management tools, including long-term populating monitoring, annual harvest levels, models, and natural resource protection regulations. As part of their management efforts, fisheries managers have learned to manage complex trade-offs. For example, increasingly they are being asked to weigh trade-offs between game, nongame, native, and nonnative species management[8].

[3] Endangered Species Act of 1973, as amended, 16 U.S.C. §§ 1531-44.

[4] See, *e.g.*, South Carolina Nongame and Endangered Species Conservation Act, S.C. Code Ann. § § 50-15-10 to -90; New Hampshire Endangered Species Conservation Act, N.H. Rev. Stat., Title XVIII, chap. 212-A; California Endangered Species Act, CA Fish & GD 3, chap. 1.5, §§ 2050 - 2116; Massachusetts Endangered Species Act, M.G.L.A. 131A; Illinois Endangered Species Protection Act, 520 Ill. Comp. Stat. (ILCS) 10/1 - 10/11.

The fisheries management approach views the fishery as a renewable resource that can be managed. It recognizes that the federal government need not protect every fish (leaving aside endangered species, which require special treatment), let alone every egg, but should instead preserve the fishery resource itself. Fisheries managers know that a certain level of cropping of fish stocks can occur without destroying a population's ability to sustain itself.

How low is too low? While the fishery science literature does not provide a definitive answer to this question, NMFS believes that a prudent rule can be established as follows: Two of the best known models in the fishery science literature find that, on average, the stock size at MSY (maximum sustainable yield) is approximately 40% of the stock size that would be obtained if fishing mortality were zero (the pristine level). . . . Also, the fishery science literature contains several suggestions to the effect that any stock size below about 20% of the pristine level should be cause for serious concern. *In other words, a stock's capacity to produce MSY on a continuing basis may be jeopardized if it falls below a threshold of about one-fifth the pristine level* (emphasis added)[9].

### Commonly Used Fishery Reference Points

Due to similarities of CWIS impacts and commercial and recreational fishing impacts, fishery management tools have been commonly applied to evaluate these impacts[57]. Regulations issued by NMFS and the Fish and Wildlife Service (FWS) incorporate the concept of "optimum yield" of a fishery, based in turn on the concept of "maximum sustainable yield" (MSY) (50 C.F.R. 600.310(c)(1)(i) (1999)). MSY is defined as "the largest long-term average catch or yield that can be taken from a stock or stock complex under prevailing ecological and environmental conditions" (*id.*). Currently, tools such as Biomass per Recruit (BPR) and spawning stock measures are more in favor than MSY.

NMFS recognizes that maximum productivity from a stock can be achieved by reducing the stock size by as much as 60% and that the population will be able to sustain or replace itself until the stock size is reduced by about 80%. Fishery managers consider removal of 70 to 80% of an unfished stock's biomass (Spawning Stock Biomass or SSB) and 65 to 80% of a stock's reproductive potential (Spawning Stock Biomass per Recruit or SSBPR) to be safe, given the compensatory reserve inherent in most fish stocks[10,11]. "Spawning Stock Biomass per Recruit" (SSBPR) is the total weight of a mature spawning stock that would be generated over the lifetime of an individual recruit[12].

When reliable estimates of the compensatory capacity of a population exist, spawner-recruit models can be used to develop more realistic and less conservative biological reference points[13]. As with the SSBPR approach, spawner-recruit analyses show that mortality due to entrainment and impingement is likely to have negligible effects on the abundance or yield of a fish population unless that population is already being fished at a level that greatly exceeds $F_{msy}$.

Biological reference points and quantitative assessment tools used in fisheries management can also be used to evaluate the likelihood that entrainment and impingement mortality will reduce the reproductive capacity of a fish population to a level that warrants management concern. Fisheries management concepts, therefore, provide scientifically sound principles for determining whether cooling-water withdrawals can cause "adverse environmental impact" to vulnerable fish populations.[5]

### Risk Assessment

No matter how sound the definition of AEI and the available assessment tools, a decisionmaking process that must decide "how much is too much" cannot escape uncertainty[15]. Assessing AEI inevitably calls for an assessment of risk to affected populations (or, for new facilities, *potentially* affected populations), to the aquatic community, and to the fishery. EPA's Ecological Risk Assessment Guidelines[16] provide a three-phase process of problem formulation, analysis, and risk characterization useful for AEI decisionmaking. The final product is a risk description that includes an interpretation of ecological adversity and descriptions of uncertainty and lines of evidence.

In short, the effect of cooling water intake structures on fisheries has many similarities to the effects of commercial and recreational fishing and associated effects (bycatch and removal of bait fish). Thus, the same general field and analytical methods developed for use in fishery management can be and have been applied to assess the effects of a CWIS on fish and shellfish in waterbodies from which cooling water is withdrawn.

## THE NEED FOR A RULE THAT MAXIMIZES NET SOCIAL BENEFIT

### Balancing Fishery Protection and Other Uses

The CWA establishes the protection of fisheries as a national goal [Clean Water Act § 101(a)(2), 33 U.S.C. § 1251(a)(2)]. Many states have likewise adopted this goal.[6] However, society has many goals for management and use of water resources, such as flood control, public water supply, agriculture, industrial water supply, and commercial and recreational fishing. Each of these uses results in impacts to fisheries, and it would be irrational to manage or regulate water resources solely for a single use such as maximizing fish production.

While any of these uses could be eliminated, to do so would result in a significant social cost. To take just one example, hydroelectric power is one of

[5] In addition to the standard fisheries management assessment tools, § 316(b) studies and other research have led to a wide range of analytical tools for assessing population-level effects. The Electric Power Research Institute recently published a catalog of analytical methods and models useful for § 316(b) decisionmaking[14].

[6] See, *e.g.*, Cal. Fish & G. Code §§ 2851, 8230 (2001); Rev. Code Wash. (ARCW) §§ 77.04.012, 77.70.160 (2001); R.I. Const. Art. I, § 17 (2001); La. Rev. Stat. 56:579.1 (2000).

the most significant in terms of volume withdrawn from a waterbody, but it also provides significant benefits such as (1) flood protection, (2) preservation of water during high-flow periods for use during low-flow periods, (3) recreational benefits, (4) increased fish habitat, (5) power production, and (6) economic development. To be sure, hydropower has deleterious effects, such as habitat fragmentation, blocking of the passage of fish, and effects on dissolved oxygen. But massive efforts are underway to mitigate these effects through impact assessments under the National Environmental Policy Act and relicensing proceedings by the Federal Energy Regulatory Commission.

Perhaps the most significant impact on fish — particularly in estuarine and marine waterbodies — is fishery exploitation[17]. In addition to the direct harvest of fish, fishery impacts occur through bycatch and bait fish removal. Another manner in which fisheries can be affected is by the deliberate introduction of nonnative species into waterbodies to promote recreational fisheries — e.g., introduction of Pacific salmon into the Great Lakes to create a recreational trout fishery and introduction of gizzard shad into reservoirs as a food source to increase sport fish populations.

In addition to water withdrawals and fishery harvests, human activities can alter fish populations in other ways. For example, land development or agricultural activities can cause sedimentation, habitat loss, and nutrient enrichment and affect dissolved oxygen levels and/or water temperature and clarity[18] and ultimately impact fisheries. Water transportation can also impact fisheries as a result of construction of navigation channels and shipping (*e.g.*, the Welland Canal, which introduced the sea lamprey into the Great Lakes, affecting the lake trout fishery) and the associated navigational use of the waterways, which can introduce exotic species in ballast water.

It is in this broader context of multiple impacts on fisheries and competing societal costs and benefits that we should approach the task of protecting fisheries from entrainment and impingement, while still providing a reliable source of electric power. Fig. 1 illustrates the three key aspects of sound § 316(b) decisionmaking. These aspects are (1) evaluation of biological conditions in the vicinity of the CWIS and assessment of the impact or potential impact to the fishery; (2) analysis of the location of the CWIS (*i.e.*, waterbody type and local aquatic community where the facility is located); and (3) CWIS design considerations.

### Biological Conditions and CWIS Impacts

Fishery management/assessment methods and tools that are available to assess fisheries and impacts from the interaction of the CWIS and the fishery were discussed earlier in this paper. Other authors — including EPA in the Economic and

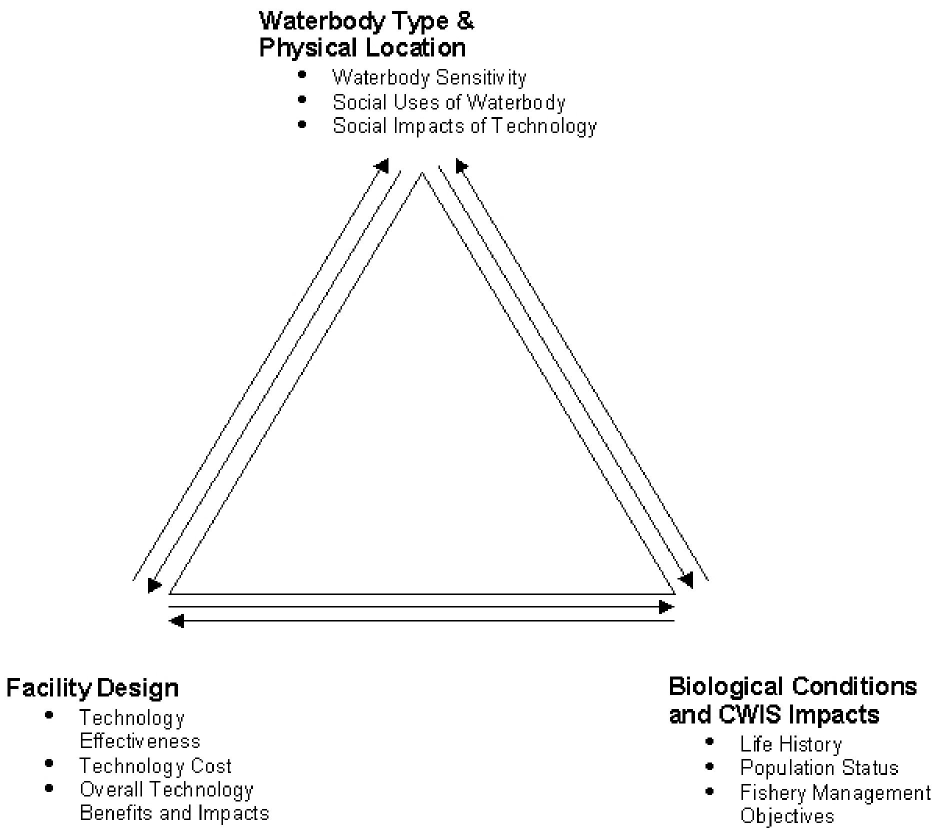

**FIGURE 1.** Key components for effective 316(b) decisionmaking.

Engineering Analyses Report developed for the Phase I § 316(b) rule[19] — have documented that very large numbers of organisms may become entrained or impinged at a single facility. If this is so, why haven't CWIS impacts been a more prominent national issue? There are a number of reasons:

- **§ 316(a) and (b) Studies** — Many states have already developed and implemented §§ 316(a) and (b) regulatory programs, including Maryland, Delaware, New York, New Jersey, Tennessee, South Carolina, California, Michigan, Ohio, Illinois, Alabama, Kentucky, Indiana, and others.[7] Studies conducted by companies located in these states (and, in some instances, independent studies conducted by the states themselves), including some long-term studies, provide a good baseline for understanding power plant fishery impacts[20]. Long-term data on one reservoir,

[7] For examples of state laws addressing impacts from cooling water intake structures, see RCSA § 22a, 430-4 (Connecticut); NJAC § 7:14A-11.6 (New Jersey); 6 NYCRR § 704.5 (New York); MRC § 26.08.03 (Maryland); 35 Ill. Admin. Code 306.201 (1998) (Illinois); 567 IAC 62.4 (455B) (Iowa); Cal. Wat. Code § 13142.5(b) (California).

Lake Wheeler, collected by the Tennessee Valley Authority[21], shed light on the relationship between long-term once-through cooling operation and the status of the fish community in the lake. Browns Ferry Nuclear (BFN) currently operates two units supported by six intake pumps with a rated total capacity of 2,312 MGD. BFN units were placed in operation between 1974 and 1977 (originally the plant supported three units). Reservoir-wide monitoring was discontinued in 1980, but cove rotenone samples were continued to provide a minimum data base on fish community in the vicinity of BFN, particularly in support of BFN's thermal variance monitoring program for the Alabama Department of Environmental Management. Cove rotenone samples have been collected annually during August and September at three sites since 1969. The data base, therefore, includes five years of pre-operational reservoir data (1969 to 1974) against which the long-term operational impacts of the plant can be compared. Details on sampling, species examined (19 species were examined, and, for each species, data were collected for three size classes: young-of-year, intermediate, and harvestable or adult), results, and analyses performed on the data are provided in TVA[21]. Although standing stock estimates for the reservoir exhibit extreme fluctuations, regression analysis revealed no significant increasing or decreasing trend for either total numbers (fish/hectare) or biomass (kg/ha) during the 30 years of monitoring.

- **Survival** — Early § 316(b) studies assumed 100% mortality to entrained organisms. Later studies, however, evaluated the survival rate of entrained organisms, many of them considering both immediate and latent mortality. EPRI recently completed a comprehensive review of entrainment mortality studies[22]. Fig. 2 presents a summary of findings demonstrating significant survival, in some cases exceeding 90%. Many of the recreationally important species had high survival rates, such as striped bass (mean survival rate 61%) and weakfish (mean survival rate 79%), while others, such as herrings and anchovies, had survival rates of approximately 25%[22,23]. Likewise, an entrainment mortality study for zooplankton at the Anclote power station in Florida demonstrated that the survival rate was quite high[24,26].
- **Stakeholder and Regulator Judgment** — Many biologists working for stakeholders, and regulatory and resource agencies as well, have judged that waterbodies where cooling water intakes operate are not impaired by entrainment and impingement. This view is reflected in the previous Administration's Clean Water Action Plan, which does not identify entrainment or impingement as a source of resource degradation[25].
- **Empirical Information** — Examples of successful fisheries in cooling ponds show that CWIS do not necessarily create adverse impact. Cooling ponds are constructed solely for the purpose of providing condenser cooling water, thereby eliminating the need for large withdrawals from a major source waterbody. Although a very high percentage of cooling pond water normally passes through the CWIS, many of these ponds support naturally reproducing fisheries[27,28,29]. While in some instances studies resulted in actions by facilities to modify their intake structures to reduce impingement or entrainment or both, or to implement offsite enhancements to avoid AEI, in most cases *no significant adverse environmental impact was identified.*

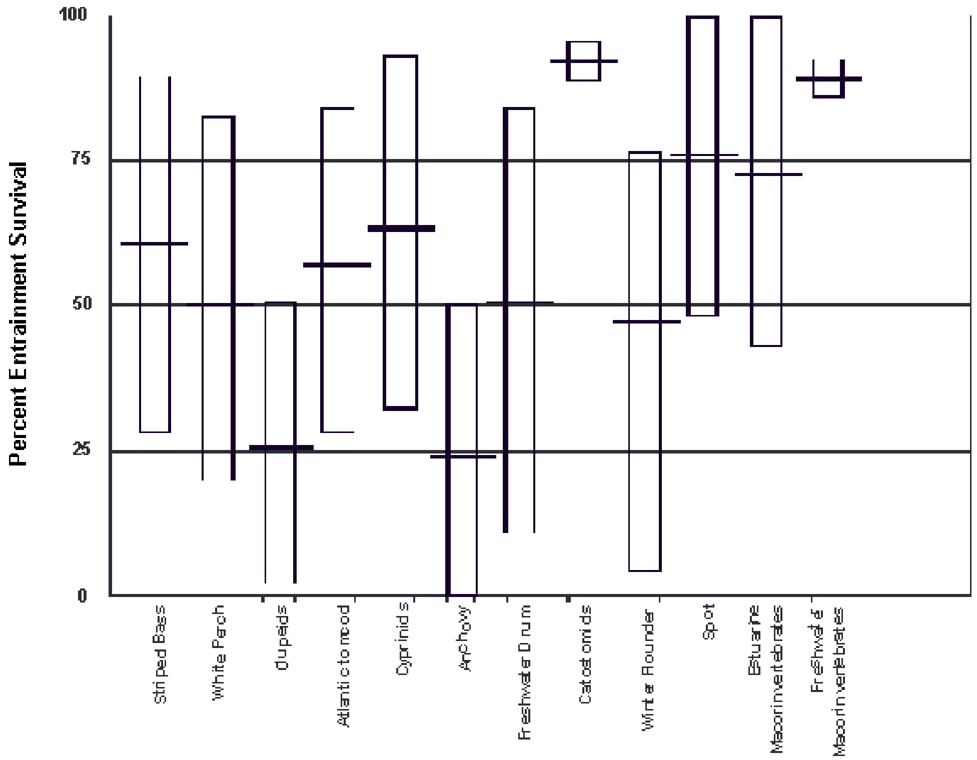

**FIGURE 2.** An illustration of the range in entrainment survival observed across various groups of fish (Source: EPRI 2000, Figure 3-1).

- **Behavioral or Life History Factors** — By virtue of their behavior or life history, many fish are able to avoid CWIS impacts[30,31,32]. For example, in freshwater many fish species lay eggs in nests or attached to substrate or vegetation, making them unavailable for entrainment. At Chalk Point, a power plant located on a tidal portion of the Patuxent River, it was initially assumed that up to 76% of each year's population in the river could be lost to entrainment. As a result of behavioral studies, however, the station determined that, due to regional movement, diurnal position in the water column, and the ability of larvae to avoid entrainment, the estimates of losses were reduced to 10 to 20%[33].
- **Compensation** — As noted by Myers[34], the concept of "compensation" is fundamental to understanding and managing biological resources. For any biological population to persist, reductions in population size caused by natural environmental fluctuations must result in increased survival, growth, or fecundity of the remaining individuals[35,36,37]. Mechanisms of compensation have been well studied in both terrestrial and aquatic systems. The compensatory response to reductions in population size is the key factor that permits fish populations to sustain themselves despite enormous natural mortality for early life stages and even intensive harvesting of adults.

Long-term research surveys have demonstrated compensation in a variety of marine, estuarine, and freshwater fish species. Field experiments in which fish population sizes are artificially manipulated have also been used to demonstrate compensation[34]. UWAG has identified approximately 50 recent scientific studies (many published in the last 10 years) demonstrating specific mechanisms responsible for compensation in a variety of fish species[38].

The National Research Council (NRC) has recognized the importance of compensation for modern fisheries management:

> Many species appear to have strongly compensatory (spawner-recruit) relationships; that is, per capita recruitment increases significantly as stock size decreases. Reference levels are now more commonly based on a % (SSBPR), but the percentage is often specified by analogy with other stocks or by using the results (of comparisons among other biological reference points). A knowledge of the compensatory capacity of the stock is necessary to define the most appropriate (biological reference points) for a stock. Even without such knowledge, however, a conservative % (SSBPR) still can be selected. (Citation omitted).[13, p. 44]

Spawner-recruit relationships of the type discussed by the NRC are used to manage two estuarine-dependent fish species, striped bass, and weakfish[39,40]. Methods discussed by the NRC can be used to incorporate the concept of compensation in management strategies for species for which spawner-recruit data are not available.

Fisheries scientists have demonstrated the importance of compensation for ensuring the continued persistence of fish populations, and fisheries managers routinely consider compensation when establishing harvesting regulations. While the precise quantification of compensation can be difficult, its occurrence cannot be disputed.

The above factors are presented not to suggest that CWIS impacts are always insignificant, but rather to put impingement and entrainment impacts in perspective. In the vast majority of cases, CWIS impacts have *not* been determined to be a substantial limiting factor for fisheries; thus, in most cases the elimination of these impacts would not be expected to substantially improve fisheries.

### Facility Design

Where adverse environmental impacts are identified, a wide range of CWIS technologies designed to reduce impacts are available, as documented in a recent EPRI report[41] and summarized in Taft 2000[42]. The EPRI report identifies a wide array of technologies available for protecting impingeable organisms, including barrier nets, angled screens, and technologies designed to take advantage of fish behavior. For protecting *entrainable* organisms, wedgewire screens, fine mesh screens and, more recently, the Gunderboom[8] have been demonstrated to be effective in certain waterbody types and for certain species, although these technologies have limitations in some waterbody types or for protection of certain species or have not yet been evaluated in a full range of waterbody conditions[43,44,45,46,47,48,49].

In addition, while not part of the CWIS, wet closed-cycle and dry cooling systems significantly reduce or eliminate the need for condenser cooling water. While some have advocated that these systems be designated as "best technology available" (BTA) for § 316(b) purposes, they can have significant negative environmental effects that would preclude their universal application. Both types of system also have significant energy requirements that reduce the efficiency and increase the fuel consumption of the generating facility. This inefficiency results in increased fuel use and air pollutant emissions, which in turn can affect water quality and fisheries by deposition of nitrogen emissions.

Wet closed-cycle cooling, which can reduce cooling water requirements by up to 98%, causes consumptive water use with fishery consequences during low-flow conditions in freshwater. Wet closed-cycle cooling towers may also be unsuitable due to their noise and vapor plumes[50]. Additionally, both wet closed-cycle and dry cooling systems have significant space requirements and aesthetic impacts. The associated increase in impervious surface (especially from dry cooling systems) can impact water quality and fisheries. Wet closed-cycle cooling systems are frequently used as components of new generation construction projects, but due to their potential environmental disbenefits, they would be a poor choice for universal BTA from a net social benefit perspective[50,51].

### Waterbody Type and Physical Location

Location considerations include characteristics such as waterbody type (marine, estuarine, riverine, or lake), the aquatic physical environment (*e.g.*, hydro- and thermodynamics, depth, and water quality conditions in the vicinity of the facility), and the local terrestrial setting (*e.g.*, urban, rural, or industrial; topography, space constraints, and proximity to facilities such as airports, historically important sites, *etc.*). Such factors directly affect the feasibility of certain CWIS technologies. In particular, use of wet closed-cycle or dry cooling systems — with their associated space requirements, noise, and aesthetic issues — can have significant effects on local communities.

[8] Fine-mesh screens have a mesh size of 0.5 to 1.0 mm. Wedgewire screens use wire with a vee- or wedge-shaped cross-section, welded to a frame to form a slotted screen. The screens are constructed in cylinders up to 7 feet in diameter, which can be attached to a common header. A Gunderboom is manufactured by mixing individual polyester fiber strands into a mat. The mat is then rolled to a specified density and pressed further by a process called needle punching, which mixes the fiber layers and improves fabric strength and durability. The permeable curtain that results can be floated and anchored in place around a cooling water intake structure.

It is therefore important that decisions balance tradeoffs in these factors to make sound decisions. The Dickerson Station on the freshwater free-flowing Potomac and the Chalk Point Station on the tidal Patuxent, both facilities located in Maryland, provide a useful example of this importance. Maryland uses the AFS fishery replacement values to quantify the value of economic losses for BTA impingement decisionmaking. These values were $11,281/yr for Dickerson and $28,450/yr (after barrier net deployment) at Chalk Point. The Department of Natural Resources estimated that the economic value of entrainment losses was approximately $1000/yr (1981 dollars) at Dickerson and had a *net present value* of $1.3 million (*i.e.*, 1989 dollar loss projected over the life of the facility) at Chalk Point. The values are low in contrast to the cost of wet closed cycle cooling, estimated to be on the order of $100 million at Chalk Point and somewhat less at Dickerson, even without considering the environmental disadvantages of this technology.

### MAKING § 316(B) DECISIONS: A PROPOSED PROCESS THAT MEETS THE NEEDS IDENTIFIED ABOVE

An approach to § 316(b) decisions that takes advantage of fishery management tools and balances multiple waterbody uses and social considerations must also be manageable and implementable from a regulatory perspective. The major components of an approach currently under development that incorporates these needs are described below. This approach establishes some distinctions between § 316(b) decisions for existing facilities and those for new facilities.

#### Decision Process for Existing Sources

The proposed approach is based on the definition of AEI presented earlier, which focuses on population- and community-level impacts and fishery use protection. It includes the elements listed below.

##### *Use of Representative Indicator Species*

Previous work has demonstrated that it is not necessary to study each and every species in a waterbody. Rather, species can be selected based on recreational or commercial importance, roles in the food chain, and/or vulnerability. Previous work has identified most of the species typically vulnerable to CWIS impacts, and site-specific screening studies can confirm the selection of species for further study as necessary.

##### *Determination of Adverse Impact*

Three alternative approaches are proposed for making § 316(b) adverse impact decisions. The first approach uses explicit criteria that are sufficiently stringent to support a decision that the facility presents no risk of adverse impact. The second approach uses a process based on the principles of EPA's risk assessment/risk management framework. The third allows decisions based on previously conducted site-specific § 316(b) studies, if it can be shown that they meet certain standards.

### Use of Screening Criteria

It is important that the decisionmaking criteria be clear and explicit to facilitate easy implementation. The criteria are not performance standards. Instead, they are designed to be well below a level that could reasonably be expected to result in AEI. This approach addresses the issue of uncertainty by setting criteria at these low thresholds. Several specific criteria being evaluated include:

- **Location.** This criterion is based on determining if a CWIS is located in a waterbody or portion of a waterbody that cannot support aquatic life at any significant level due to poor water quality, such as anoxia, or lack of habitat. For example, if the CWIS withdraws its intake water from an anoxic zone which cannot support impingeable and entrainable organisms important to the fishery, it would be very unlikely to result in AEI.
- **Facility design.** If a facility employs a CWIS which is designed or has features to minimize impingement and/or entrainment, or makes use of technologies such as wet closed-cycle cooling, it would present no appreciable risk of AEI. In this situation, the technology must be demonstrated to be effective. If the technology is known to be effective only for impingement, for example, then the issue of entrainment will still need to be assessed.
- **Percentage of waterbody used.** Use of this criterion is suggested for entrainable organisms in smaller waterbodies such as freshwater rivers, lakes and reservoirs. A criterion value of 5% (or less) of the 90% exceedance flow of a river or of the volume of the biological zone of influence[9] in a lake or reservoir, measured when entrainable life stages of RIS are present, is proposed. This approach essentially is based on a 95% protection standard, which is believed to be adequately protective for freshwater locations.
- **Biological criteria.** The low-risk biological criteria being evaluated again are limited to use in freshwater rivers, reservoirs, and lakes other than the Great Lakes. Criteria of 5% (or less) loss of a non-harvested species and 1% (or less) loss of a harvested species are being considered as values that would generally

[9] The biological zone of influence is the zone within a waterbody that is occupied by an RIS. For freshwater rivers the biological zone of influence for RIS entrainable life stages is the portion of the cross-sectional flow of the river occupied by the RIS where the river flows past the CWIS. If an RIS is found primarily along the shoreline, the biological zone of influence is the sum of the flow along the shorelines on both sides of the river. For smaller freshwater lakes and reservoirs and controlled-flow rivers that have lake-like characteristics, the biological zone of influence is the volumetric area occupied by the RIS during the time when the RIS is vulnerable to entrainment. Reservoirs and large rivers with controlled flow may have either riverine or lake-like characteristics, several types of spawners, and perhaps disproportional distribution of habitat (upstream versus downstream of the plant). For such waters the more appropriate of the above two criteria must be determined and applied.

pose no risk of adverse impact. These values are low compared to generally allowable fishery harvest management levels. Other biological criteria, also being considered, would take advantage of well-designed long-term monitoring programs and measures such as the multi-metric criteria developed by Duke Power and TVA, in use at many southern reservoirs.

### *Use of Risk Assessment and Risk Management Principles*

A second method of AEI decisionmaking involves use of EPA's ecological risk assessment/risk management guidelines. This approach includes active stakeholder participation, in which natural resource managers and interested members of the public identify populations of special interest for assessing the potential impact of the CWIS. These form the basis of the next step, which is identification of appropriate methods and analytical approaches. Finally, study endpoints are established to allow easy AEI decisionmaking after data collection. The process can address uncertainty by balancing comprehensiveness of study design, use of fishery information for species of concern, and modeling assumptions.

### *Use of Previously Conducted § 316(b) Studies*

This approach makes use of the extensive § 316(b) studies already conducted at many facilities. Any studies that are not reasonably current would have to be evaluated to ensure that the studies are representative of the current facility design and biological conditions and that the data collection methods and analytical tools remain valid. In particular, sufficient information must be provided to show that the populations examined continue to be appropriate in terms of fishery management objectives. The objective of this approach is to take full advantage of the previous investment in data collection and evaluation conducted by regulators. Fishery managers and other stakeholders would be able to participate in the evaluation through the NPDES process.

The above three decisionmaking approaches could be used independently or in combination. For example, screening criteria could be used initially to provide focus to determine appropriate RIS, with the final decisionmaking done using the risk-based approach. Finally, the decision process for existing facilities would incorporate three additional features: using cost-benefit analysis to maximize net benefit, allowing "environmental enhancements" in appropriate circumstances, and reviewing BTA determinations if new information showed that circumstances had changed.

### *Maximum Net Benefit*

If the decisionmaking process outlined above shows that an existing CWIS is creating, or will create, an appreciable risk of AEI, then the decisionmaker must decide what is "best technology available" or BTA to "minimize" AEI. UWAG's economic consultant advises that the most rational way to make this decision is to choose the technology that maximizes net benefits (that is, benefits minus costs). To use this approach, the permit applicant would have to identify all reasonably available intake structure technologies that would reduce the impact to the aquatic community and be feasible at the site. The applicant would also estimate the costs and benefits of each such technology, including the impacts of the CWIS on aquatic biota, in addition to the monetary costs of construction and operation, energy costs, and environmental costs such as air pollution, aesthetics, and land use. Summing the costs and benefits for each "available" technology, the permittee would choose as "best" the one that had the highest net benefit. Industry believes that cost-benefit analyses suitable for BTA selection can be developed based on existing tools and methods, such as by adopting some features of EPA's BEN model for evaluating the benefits of violating environmental laws or of the methods used to evaluate natural resource damages[52].

### Environmental Enhancements

"Environmental enhancements" are actions taken by a facility determined to cause AEI (or a facility that wishes to settle a dispute over its permit) to compensate for the CWIS losses to the affected RIS species rather than install a CWIS technology. Environmental enhancements — such as wetlands creation or fish stocking — are one means of compensating for CWIS losses. In some cases, the *most* limiting factor for the aquatic populations is not the CWIS, but rather (1) low dissolved oxygen as a result of nutrient enrichment or (2) lack of habitat for spawning and nursery functions[53]. In such cases, by investing dollars addressing the most limiting environmental factors, the facility may spur a more significant recovery to the population than could be achieved through installation of a CWIS technology. Such actions, as long as they are directly related to the fishery, can result in a greater net social benefit than installation of BTA. Enhancements have been used effectively at a number of stations, including Salem, John Sevier, and Chalk Point. Florida Power Corporation's Crystal River fish hatchery is another successful enhancement program. Such environmental enhancements are not "intake technologies" (and therefore cannot be "BTA" nor be required by authority of § 316(b)), but the § 316(b) regulatory framework is flexible enough to allow them to be used, if offered by the permit applicant. They can be employed as a cost-effective means of addressing adverse environmental impact, potentially resulting in environmental benefits greater than use of BTA alone.

### Periodic BTA Review

Once an existing facility has gone through the process of determining that the CWIS is BTA, the BTA status would need to be revisited at the time of permit renewal if the regulatory agency had information showing that the previous studies were no longer valid (for example, that biological conditions had changed). Factors that might result in a change of BTA status would include modifications to the CWIS design or operation or significant changes in the waterbody.

### Decision Process for New Facilities

The process described above is for existing facilities. For new facilities, a "Two Track" approach has been proposed that would allow a company seeking to build a new facility that will require use of surface cooling water either (1) to commit to a highly protective (indeed often *over*-protective) technology at the outset or (2) to engage in a site-specific analysis to determine whether the intake would create an appreciable risk of AEI and, if so, what would be the BTA for the site.

#### *Track 1: The "Fast Track"*

Track 1, the "Fast Track," would allow the applicant to commit to one of the following highly protective technologies, in return for expedited permitting without the need for pre-operational or operational studies in the source waterbody by using one of two options:

- Option 1: Employ a technology that limits intake flow to the flow that would be required by wet closed-cycle cooling for a given amount of generation at that site and design the average approach velocity (measured in front of the intake screens or the opening to the cooling water intake structure) to be no more than 0.5 ft/s; or
- Option 2: Employ a technology that will achieve a level of protection from impingement and/or entrainment that is reasonably consistent with Option 1. This option is intended to permit facilities to use either standard technologies, or new ones, that have been demonstrated to be effective for the species, type of waterbody, and flow volume proposed for their use. Examples of candidate technologies include:

a. Wedgewire screens, where there is constant flow, as in rivers;
  b. Traveling fine mesh screens with a fish return system designed to minimize entrainment and impingment mortality; and
  c. Gunderbooms, at sites where they would not be rendered ineffective by high flows or fouling.

"Reasonably consistent with" means that an acceptable alternative technology should provide a level of protection within the range expected under Option 1 achieved by flow reductions associated with wet closed-cycle cooling and a 0.5 ft/s approach velocity for the type of waterbody on which the facility is to be sited. Use of highly protective technologies should eliminate the need for periodic BTA review.

The effectiveness of wet closed-cycle cooling is well documented. The other technologies listed above promise a level of protection reasonably consistent with that of wet closed-cycle cooling. To prevent impingement, the Gunderboom is designed to have a low approach velocity (almost unmeasurable) and uses a very fine mesh to provide entrainment protection[42]. Wedgewire screens are designed to minimize entrainment and impingement through a combination of small slot width (0.5 to 2 mm) and an approach velocity of less than 0.5 ft/s[42,54].

For fine mesh screens, the survival of fish collected on the screens is species- and life- stage-specific[41,42]. Survival of many species can be very high, exceeding 90% even at velocities above 0.5 ft/s. As for entrainment, again the effect of fine mesh screens varies by species, but the data indicate that, if control mortality is taken into account, fine mesh screens can reduce entrainment mortality by 90% or more for some species. Other species, such as bay anchovy, have a high mortality both naturally and after encountering fine mesh screens. Nevertheless, given the present state of knowledge, it is reasonable to include fine mesh screens (with a properly designed fish return system) as a candidate technology for some sites that can reduce overall losses to a level (*i.e.*, 90% or better) reasonably consistent with wet closed-cycle cooling.

Option 2 of Track 1 encourages alternative or innovative intake structure technologies. A proponent of a new alternative technology would conduct a laboratory or site-specific study appropriate for the waterbody type and species of concern prior to employment of the technology. If the demonstration was successful, after the facility deployed the new technology, monitoring would be conducted as appropriate to validate performance.

At a few sites, there could potentially be unusual species-specific circumstances in which Fast Track technologies meeting the above criteria would not be sufficient to avoid AEI. While the number of such sites is likely to be very small, the evaluation process should give permit writers the authority to require additional protective measures *if* the permitting agency has information to support a finding that exceptional conditions exist such that the proposed facility could affect one or more populations in a way that would not be prevented by other federal or state requirements (such as the Endangered Species Act) and thus has the potential to cause AEI.

### *Track 2: A More Tailored Approach*

Track 2 of the proposed Two Track approach is similar to the decisionmaking process for existing facilities summarized above. It differs in that Track 2 for new facilities can make use only of predictive fishery management tools, rather than retrospective ones. Track 2 would be for facilities that wished to pursue use of a less stringent BTA.

In these cases the applicant could evaluate AEI using the risk screening criteria or the risk assessment/risk management AEI evaluation methods for existing sources. For the population percent reduction criteria, source waterbody type, data availability and assessment, and analytical tool availability will determine the difficulty of predicting impingement rates in a sufficiently quantitative manner. Where this cannot be done, new facilities will need to plan for some kind of technology to protect fish from impingement.

The Two Track decision process, then, is both efficient and flexible, and it has one very important advantage: it avoids worsening the already-present “energy crisis” now affecting California and possibly soon other states[55,56,57]. Track 1, the Fast Track, is available for speeding new generating facilities online in parts of the country where they are needed most, in return for a commitment to highly (often overly) protective intake structure technology, and also encourages innovative technologies. Track Two allows a close look at the features of any proposed site and avoids arbitrary, less efficient, restrictions.

### CONCLUSION

The Clean Water Act requires that cooling water intake structures minimize, where it exists, "adverse environment impact." In order to be able to determine whether AEI exists or is threatened, and if it exists to decide how to minimize it, one must first have a definition. The definition needs to ensure the protection of living resources. And the process for "minimizing" AEI needs to strike a balance among competing social needs.

Tools are available today to accomplish both these goals. The science of fisheries management provides concepts (like maximum sustainable yield), tools (like biological modeling), and knowledge (such as knowledge of how fish populations compensate for losses) that will allow cooling water users and regulatory agencies to make sound § 316(b) decisions that will protect the living fishery resources. Cost-benefit analysis, drawing on experience of calculating the benefits of environmental violations and natural resource damages, provides a tool for choosing an intake technology that maximizes the net benefits to society.

Given a workable definition of AEI and the tools to assess and minimize it, one needs, finally, a decisionmaking process that allows the tools to be used appropriately. The electric utility industry has proposed such a process, one that provides both the opportunity to bring new generating plants online quickly, in return for installing highly (often overly) protective intake technology, and the flexibility to look closely at site characteristics when assessing the risk of AEI, and taking advantage of site characteristics as appropriate to concurrently protect the environment and produce energy efficiency.
